# Effect of facilitation of local maternal-and-newborn stakeholder groups on neonatal mortality: a cluster randomised trial

**DOI:** 10.1186/1472-6963-14-S2-O28

**Published:** 2014-07-07

**Authors:** Lars Åke Persson, Nga Nguyen T, Mats Målqvist, Hoa Dinh Thi Phuong, Leif Eriksson, Lars Wallin, Katarina Selling, Huy Tran Q, Duc Duong M, Uwe Ewald

**Affiliations:** 1 International Maternal and Child Health, Department of Women’s and Children’s Health, Uppsala University, Uppsala, Sweden; 2 Vietnam-Sweden Uong Bi General Hospital, Uong Bi, Viet Nam; 3 Hanoi School of Public Health, Hanoi, Viet Nam; 4 School of Education, Health and Social Studies, Dalarna University, Falun, Sweden; 5 Ministry of Health, Hanoi, Viet Nam; 6 Provincial Health Bureau, Quang Ninh Province, Viet Nam

## Background

Facilitation of local women’s groups may reportedly reduce neonatal mortality. It is not known whether facilitation of groups composed by local healthcare staff and politicians can improve perinatal outcomes. We hypothesized that facilitation of local stakeholder groups would reduce neonatal mortality (primary outcome) and improve maternal, delivery and newborn care indicators (secondary outcomes) in Quang Ninh province, Vietnam. Trial registration: Current Controlled Trials ISRCTN44599712.

## Material and methods

In a cluster-randomised design, 44 communes were allocated to intervention and 46 to control. Laywomen facilitated monthly meetings during 3 years in groups composed by healthcare staff and key persons in the communes. A problem-solving approach was employed. Births and neonatal deaths were monitored, and interviews were performed in households of neonatal deaths and of randomly selected surviving infants. A latent period before effect is expected in this type of intervention, but this timeframe was not pre-specified.

## Results

Neonatal mortality rate (NMR) from July 2008 to June 2011 was 16.5/1000 (195 deaths per 11818 live births) in the intervention communes and 18.4/1000 (194 per 10559 live births) in control communes (adjusted odds ratio 0.96 [95% CI 0.73-1.25]). There was a significant downward time trend of NMR in intervention communes (p=0.003) but not in control communes (p=0.184). No significant difference in NMR was observed during the first two years (July 2008 to June 2010) while the third year (July 2010 to June 2011) had significantly lower NMR in the intervention arm; adjusted odds ratio 0.51 [95% CI 0.30-0.89]. Women in intervention communes more frequently attended antenatal care (adjusted odds ratio 2.27 [95% CI 1.07-4.8].

## Conclusions

A randomised facilitation intervention with local stakeholder groups composed by primary care staff and local politicians working for three years with a perinatal problem-solving approach resulted in increased attendance to antenatal care and reduced neonatal mortality after a latent period.

